# Assessment of Structural Barriers and Racial Group Disparities of COVID-19 Mortality With Spatial Analysis

**DOI:** 10.1001/jamanetworkopen.2022.0984

**Published:** 2022-03-04

**Authors:** Qinyun Lin, Susan Paykin, Dylan Halpern, Aresha Martinez-Cardoso, Marynia Kolak

**Affiliations:** 1 Center for Spatial Data Science, The University of Chicago, Chicago; 2 Department of Public Health Sciences, The University of Chicago, Chicago

## Abstract

**Importance:**

Although social determinants of health (SDOH) are important factors in health inequities, they have not been explicitly associated with COVID-19 mortality rates across racial and ethnic groups and rural, suburban, and urban contexts.

**Objectives:**

To explore the spatial and racial disparities in county-level COVID-19 mortality rates during the first year of the pandemic.

**Design, Setting, and Participants:**

This cross-sectional study analyzed data for all US counties in 50 states and the District of Columbia for the first full year of the COVID-19 pandemic (January 22, 2020, to February 28, 2021). Counties with a high concentration of a single racial and ethnic population and a high level of COVID-19 mortality rate were identified as concentrated longitudinal-impact counties. The SDOH that may be associated with mortality rate across these counties and in urban, suburban, and rural contexts were examined. The 3 largest racial and ethnic groups in the US were selected: Black or African American, Hispanic or Latinx, and non-Hispanic White populations.

**Exposures:**

County-level characteristics and community health factors (eg, income inequality, uninsured rate, primary care physicians, preventable hospital stays, severe housing problems rate, and access to broadband internet) associated with COVID-19 mortality.

**Main Outcomes and Measures:**

Data on county-level COVID-19 mortality rates (deaths per 100 000 population) reported by the US Centers for Disease Control and Prevention were analyzed. Four indexes were used to measure multiple dimensions of SDOH: socioeconomic advantage index, limited mobility index, urban core opportunity index, and mixed immigrant cohesion and accessibility index. Spatial regression models were used to examine the associations between SDOH and county-level COVID-19 mortality rate.

**Results:**

Of the 3142 counties included in the study, 531 were identified as concentrated longitudinal-impact counties. Of these counties, 347 (11.0%) had a large Black or African American population compared with other counties, 198 (6.3%) had a large Hispanic or Latinx population compared with other counties, and 33 (1.1%) had a large non-Hispanic White population compared with other counties. A total of 489 254 COVID-19-related deaths were reported. Most concentrated longitudinal-impact counties with a large Black or African American population compared with other counties were spread across urban, suburban, and rural areas and experienced numerous disadvantages, including higher income inequality (297 of 347 [85.6%]) and more preventable hospital stays (281 of 347 [81.0%]). Most concentrated longitudinal-impact counties with a large Hispanic or Latinx population compared with other counties were located in urban areas (114 of 198 [57.6%]), and 130 (65.7%) of these counties had a high percentage of people who lacked health insurance. Most concentrated longitudinal-impact counties with a large non-Hispanic White population compared with other counties were in rural areas (23 of 33 [69.7%]), included a large group of older adults (26 of 33 [78.8%]), and had limited access to quality health care (24 of 33 [72.7%]). In urban areas, the mixed immigrant cohesion and accessibility index was inversely associated with COVID-19 mortality (coefficient [SE], –23.38 [6.06]; *P* < .001), indicating that mortality rates in urban areas were associated with immigrant communities with traditional family structures, multiple accessibility stressors, and housing overcrowding. Higher COVID-19 mortality rates were also associated with preventable hospital stays in rural areas (coefficient [SE], 0.008 [0.002]; *P* < .001) and higher socioeconomic status vulnerability in suburban areas (coefficient [SE], –21.60 [3.55]; *P* < .001). Across all community types, places with limited internet access had higher mortality rates, especially in urban areas (coefficient [SE], 5.83 [0.81]; *P* < .001).

**Conclusions and Relevance:**

This cross-sectional study found an association between different SDOH measures and COVID-19 mortality that varied across racial and ethnic groups and community types. Future research is needed that explores the different dimensions and regional patterns of SDOH to address health inequity and guide policies and programs.

## Key Points

**Question:**

How do the associations between structural factors and COVID-19 mortality help explain the disproportionate outcomes experienced by different racial and ethnic groups?

**Findings:**

In this cross-sectional study of 3142 counties in 50 US states and the District of Columbia, the associations between different measures of social determinants of health and COVID-19 mortality varied across racial and ethnic groups (Black or African American, Hispanic or Latinx, and non-Hispanic White populations) and different community types (rural, suburban, and urban areas).

**Meaning:**

Findings from this study suggest the need for future research that addresses health inequity and guides policies and programs by further exploring the different dimensions and regional patterns of social determinants of health.

## Teaser

This cross-sectional study uses spatial analysis to evaluate social determinants of health and COVID-19-related mortality rates among Black, Hispanic, and White populations living in rural, suburban, and urban communities.

## Introduction

In the US, the COVID-19 pandemic has disproportionately affected racial and ethnic minority groups.^1^ Nationally, COVID-19 has been associated with higher infection and mortality rates in American Indian or Alaska Native, Black, and Hispanic or Latinx communities across state and regional levels.^2^ This pattern is consistent with racial and ethnic minority groups bearing a disproportionate burden of health inequalities, which are associated with morbidity and mortality, in the US today.^3^

Evidence shows that social determinants of health (SDOH), the structural conditions that characterize where people live, work, and play, are substantial factors in racial and ethnic health disparities, including disparities in COVID-19 infection and mortality rates.^4,5,6,7^ Race is primarily a social construct based on nationality, ethnicity, phenotype, or other markers of social difference; it has little impact on genealogical or biological differences. Instead, racial disparities in health largely emanate from the inequitable access to social, economic, and physical or built environmental conditions resulting from racism in the US. Specifically, racism interacts with and exists within societal structures and systems to shape the major SDOH, including the housing, labor, and credit markets as well as the education, criminal justice, economic, and health care systems.^8^ This inequity occurs at both interpersonal and structural levels, positioning stigmatized racial and ethnic minority populations at greater risk for poor outcomes.

There is a demonstrated need to consider the role of these structural factors of inequality in understanding health outcome disparities.^9^ In the context of the COVID-19 pandemic, researchers have identified structural racism as a factor in the disproportionate burden on Black individuals,^10^ Indigenous peoples,^11^ and Hispanic or Latinx communities,^12^ yet relatively few analyses have explicitly reported an association between SDOH and COVID-19 mortality rates. In this cross-sectional study, we used measures of SDOH in US counties with the highest percentages of racial and ethnic minority groups as well as the highest mortality rates as a proxy to examine whether SDOH were associated with COVID-19 mortality across racial and ethnic minority groups and communities.

**Figure 1.**
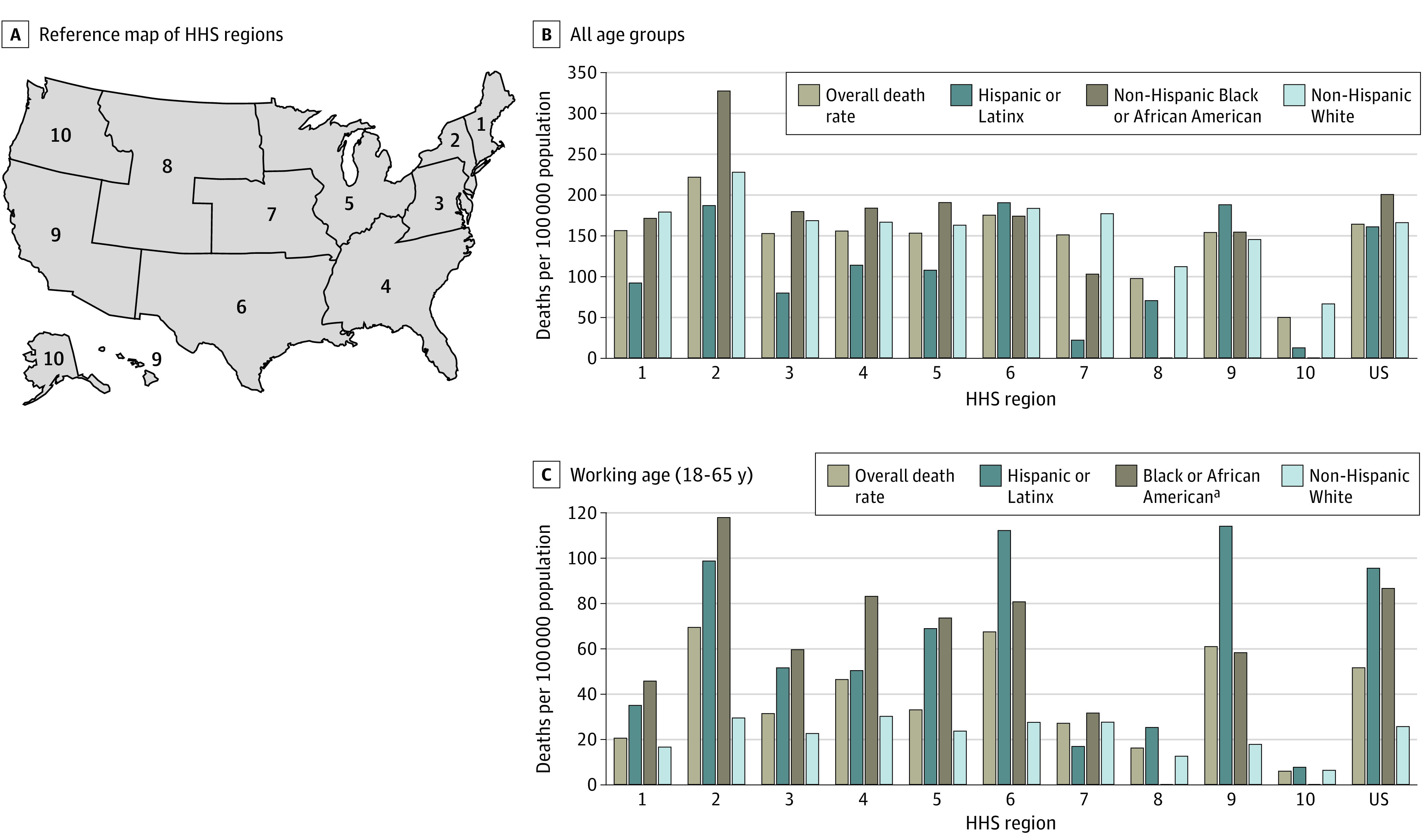
COVID-19 Death Rates by American Community Survey Race and Ethnicity and US Department of Health and Human Services (HHS) Region and by Age for Hispanic or Latinx, Black or African American, and non-Hispanic White Populations Death rates were calculated as the number of deaths for each racial or ethnic group reported from January 22, 2020, to February 28, 2021, divided by the population for that HHS region and multiplied by 100 000. ^a^Estimates for Black or African American communities were potentially conservative because the Census data on age by race and ethnicity are not disaggregated by Hispanic or non-Hispanic origin.

One challenge in quantifying the implications of structural racism for COVID-19 outcomes is that the US Centers for Disease Control and Prevention (CDC) does not report race-disaggregated COVID-19 data below the state level. The Modifiable Areal Unit Problem in geography highlights issues of different findings produced by varied levels of aggregation,^13^ suggesting that state-level estimates can mask heterogeneous population patterns and make community-level impacts difficult to evaluate.^14^ Initial explorations of CDC data underscore this challenge. Figure 1 illustrates provisional CDC data aggregated by the US Department of Health and Human Services (HHS) region,^15^ the most granular level at which both race- and age-disaggregated data are currently available. Data in most HHS regions show higher death rates for Hispanic and non-Hispanic Black populations compared with non-Hispanic White populations. Clearer disparities are shown when comparing death rates for the working-age population aged 18 to 65 years. The associations between these rates are not uniform across HHS regions, indicating racial and spatial heterogeneity at the regional scale. This heterogeneity likely persists below the regional and state levels; eFigure 1 in the Supplement provides additional data at the state level.

County-level data provide more fine-grained information, but race- or age-disaggregated COVID-19 data at this scale are, to our knowledge, unavailable nationally. Researchers address such issues of limited data in varying ways, such as comparing counties with high proportions of a racial and ethnic group^16^ or high mortality^17^ with all other counties. Although these approaches highlight the disproportionate burden experienced by racial and ethnic minority groups and the potential contribution of SDOH, these approaches miss the potential heterogeneity in different groups in different contexts (eg, rural vs urban areas) as well as fail to account for the contagious nature of COVID-19.

This contagiousness requires analysis to make the spatial effects explicit^18^ given that the virus is highly transmissible between individuals (spatial dependence) and has exhibited uneven spread across geographic areas and populations (spatial heterogeneity).^19^ An exploratory spatial data analysis (ESDA) approach helps address these research challenges.^20,21^ Using ESDA, we explored the spatial and racial disparities in county-level COVID-19 mortality rates across national-scale data sets during the first year of the pandemic to gain insights into the dimensions of structural racism closely associated with COVID-19 mortality. The spatial perspective allowed us to examine the potential heterogeneity in different racial and ethnic groups across rural, suburban, and urban contexts and to explicitly account for contagious outcomes across county borders.

## Methods

This observational, cross-sectional study included all counties in 50 states and the District of Columbia (N = 3142), drawing on county-level COVID-19 mortality data reported by the CDC from January 22, 2020, to February 28, 2021. Racial and ethnic and other demographic data as well as other SDOH measures were sourced from publicly available data sets. The study was deemed exempt from review and the requirement for informed consent by the institutional review board at The University of Chicago because it used publicly available, deidentified data. We followed the Strengthening the Reporting of Observational Studies in Epidemiology (STROBE) reporting guideline.

The primary outcome was county-level COVID-19 mortality rates. We included the first 2 months in 2021 because the COVID-19 vaccines became effective after 2 weeks of being fully vaccinated^22^ and the outcome variable of mortality may have some further lag effect. County mortality data were sourced from the CDC COVID Data Tracker^23^ (N = 3142). We calculated the number of COVID-19 deaths per 100 000 population using the 2019 American Community Survey (ACS) 5-year estimates. This number served as the outcome variable in the regression analysis.

### Racial and Ethnic Groups

We focused on the 3 largest racial and ethnic groups in the US according to the 2019 ACS 5-year estimates: Black or African American, Hispanic or Latinx, and non-Hispanic White populations. Race and ethnicity were self-identified in the ACS. We calculated the percentage of the adult population (aged ≥18 years) for each racial and ethnic group for each county.

### Social Determinants of Health and Structural Factors

To capture multiple dimensions of SDOH, we used 4 indexes developed by Kolak et al,^5^ which were extracted from an adapted socioecological model of health that spans individual, interpersonal, organizational, and community metrics. Using a principal component analysis, Kolak et al^5^ created 4 indexes to account for 71% of the variance in their SDOH model, which included 15 variables in the continental US. Specifically, the 4 indexes were (1) the socioeconomic advantage index, which is dominated by socioeconomic status factors and in which a low value is characterized by a high percentage of the population living in poverty, with a racial and ethnic minority status, without a high school diploma, and without health insurance; (2) the limited mobility index, in which a low value is characterized by a high percentage of older adults (≥65 years) and people with disabilities; (3) the urban core opportunity index, in which a high value reflects a highly urbanized population with more opportunities along with a high per-capita income and high living costs such as a high rent burden; and (4) the mixed immigrant cohesion and accessibility index, in which a lower value is characterized by more immigrant populations with traditional family structures and multiple accessibility stressors such as crowded housing and lack of health insurance. These measures expanded beyond a single dimension of socioeconomic disadvantages and were found to have a validated association with premature mortality in Chicago, Illinois.^5^ For this analysis, we aggregated Census tract–level indexes to the county level.

Additional variables for measuring structural factors were included in this study to capture potential social factors that were not covered in the 4 indexes but were crucial to COVID-19 mortality and provide more contextual information to help in understanding and interpreting the patterns in the 4 indexes. As principal components, the 4 SDOH indexes are orthogonal to each other by construction to reflect most information in multiple variables, but they may be difficult to interpret at the same time owing to the compression of correlated variables. Thus, we included specific variables to help with this interpretation. In particular, we included age distribution (ie, working age vs age ≥65 years), rural-urban context, and multiple community health factors (eTable 1 in the Supplement). For the rural-urban context, we used a classification system based on the 2010 Rural-Urban Commuting Area codes, which classified Census tracts as rural, suburban, or urban according to their primary Rural-Urban Commuting Area codes.^24^ Counties with more than 50% urban tracts were classified as urban counties, counties with more than 50% suburban tracts as suburban, and counties with more than 50% rural tracts as rural.

Additional community-level health factors were identified from theoretical evidence and literature on neighborhood characteristics and COVID-19 mortality^25,26^ using a participatory design approach.^14^ These factors included income inequality, uninsured rate, primary care physicians, preventable hospital stays, severe housing problems rate, and access to broadband internet. Preventable hospital stays were measured as the rate of hospital stays for ambulatory care–sensitive conditions per 100 000 Medicare enrollees. We considered 2 additional context covariates: group quarter population rate (per 100 000) and delay in mask mandates; for the second variable, we counted the number of days that a state-level mask mandate was initiated after the occurrence of more than 200 new confirmed cases per 100 000 populations per 14 days. eTable 1 in the Supplement provides details of how these variables were measured.

### Statistical Analysis

Because no previous studies have explored the study question (How do the associations between structural factors and COVID-19 mortality help explain the disproportionate outcomes experienced by different racial and ethnic groups?) at the national scale, we adopted an exploratory approach with multiple streams of inquiries to identify patterns that we did not expect to see. We started with colocation analysis and visual analytics to identify counties that were severely affected by COVID-19 during the study period (January 22, 2020, to February 28, 2021), particularly areas in which each racial or ethnic group in the study was experiencing high COVID-19 mortality rates. We then analyzed the potential community characteristics of these areas that are statistically associated with their high mortality rates. This initial step allowed us to refine our understanding of the data-generating processes, which were then operationalized in the spatial regression using raw data. In particular, we deployed spatial regime regression models to examine the associations of multiple aspects of structural factors with the county-level COVID-19 mortality.

All statistical tests were 2-sided with a significance level of α = .05, with Bonferroni correction for multiple comparisons. All statistical analyses were performed using R, version 4.0.2 (R Foundation for Statistical Computing), and GeodaSpace, version 1.2 (GeodaSpace).

#### Colocation Analysis

We used colocation analysis to identify counties with consistently high COVID-19 death rates that had a high proportion of residents of a particular racial or ethnic group compared with other counties. Colocation analysis is rooted in the association between geographic locations and economic activities and processes^27^ and has been applied across various fields to study spatial associations between different events.^28,29,30^ For the present analysis, if a county (1) had mortality rates in the top quintile for 100 days or more over the year (the 100 days did not need to be consecutive) and (2) was in the top quintile of adult populations of a particular racial or ethnic group, then we labeled it as a concentrated longitudinal-impact county. The top quintile of population was different for each racial or ethnic group: 14.2% for the Black or African American group, 10.2% for the Hispanic or Latinx group, and 94.5% for the non-Hispanic White group.

Although consecutive-day hot spots (ie, mortality rates in the top quintile for consecutive days) would likely indicate a more severe COVID-19 impact, the number of counties that met this selection criteria would be small (34 for Black or African American, 46 for Hispanic or Latinx, and 3 for non-Hispanic White groups), making interpretation difficult. Previous research has used similar approach. For example, from March 8 to July 15, 2020, Oster et al^31^ identified 26% of counties (n = 818, representing 80% of the US population) that were considered hot spots for COVID-19 transmission for at least 1 day, wherein the median number of days (not necessarily consecutive) that a county met the hot spot criteria was 10.

We summarized mortality rates, SDOH indexes, and structural factors for each concentrated longitudinal-impact county and the remaining non–concentrated longitudinal-impact counties for comparison. For each measure included (ie, mortality rates, SDOH indexes, and structural factors), we implemented analyses of variance to test for statistically significant differences in SDOH indexes and structural factors across concentrated longitudinal-impact counties using Bonferroni correction for multiple comparisons.

#### Visual Analytics

Because race-disaggregated COVID-19 data are not available below the county level for all states, we used dot density mapping as a visual analytic technique to illustrate where racial or ethnic groups tended to live at the Census tract level and where COVID-19 death data were recorded at the county level. Such dot maps have recently been widely used to delineate spatial distributions of discrete geographic features, such as social demographic characteristics.^32,33,34^ In the present analysis, the dot density technique visualized the within-county variation of racial and ethnic composition according to the ACS census estimate using 1 dot to represent 120 people by racial and ethnic group assorted by Census tract level. Each map panel illustrated a different racial and ethnic group, which was visualized as a dot density map. These dots were then assigned a color scheme according to the number of days that county had a high COVID-19 mortality rate (ie, in the top quintile of the national distribution). Using this method, we assumed that mortality was distributed equally across all racial and ethnic groups within the same county; this was a conservative approach given that disparity is known to exist even within counties.^4,26^ At the same time, population typologies (density and racial and ethnic composition) can be visually highlighted so that variations between counties are made more apparent, although within-county variation of mortality is hypothesized as a conservative estimate of equal distribution. We also used choropleth maps with fixed bins to geovisualize the number of days that each county had a high death rate, with the concentrated longitudinal-impact counties highlighted.

#### Spatial Regime Regression Models

We hypothesized that distinct patterns of concentrated longitudinal-impact counties would emerge across urban, suburban, and rural contexts. We modeled urban, suburban, and rural counties as different spatial regimes, reflecting different processes through which SDOH dimensions were associated with COVID-19 mortality. The SDOH as independent variables may uniquely be associated with the COVID-19 mortality outcome in different spatial regimes. Each regime was then characterized by different values of regression coefficients in such spatial regime models.^35^ This approach is often adopted in regional science to capture structural instability.^36,37,38^

Considering the contagious nature of COVID-19, we assumed that mortality across counties may not be independently distributed, which is a core assumption of traditional statistical analysis. We tested for spatial autocorrelation using a Moran *I* statistic and found that COVID-19 mortality rates had a statistically significant spatial autocorrelation (Moran *I* = 0.34; pseudo-*P* = .001). This result invalidated traditional statistical analysis (including log-likelihood ratios), which assumes an independent, identically distributed phenomenon. We thus adopted spatial regression to account for spatial autocorrelation. Such spatial econometric approaches have been adopted to study spatial epidemiologic processes^39,40^ and are increasingly common in relevant policy and public health literature,^41,42,43^ including COVID-19 studies at smaller regional scales.^44,45,46^ We operationalized a second-order queen contiguity spatial weight in spatial models to model the spatial interdependence; that is, we allowed an interaction between a county and its neighbors as well as the neighbors of its neighbors (eFigure 2 in the Supplement). We excluded some variables to avoid multicollinearity issues with the SDOH indexes.

We started with the ordinary least-squares (OLS) estimates, which do not consider spatial autocorrelation to observe collinearity among all factors. First, we included 4 SDOH indexes because they were orthogonal to each other by construction and reflected most information across various SDOH dimensions. Second, we added community characteristics to the model to capture factors that were associated with COVID-19 but were not covered by the 4 SDOH indexes. Variables that were correlated with the 4 indexes were excluded to avoid multicollinearity, including age distribution, income inequality, uninsured rate, primary care physicians, and severe housing problems. Third, we fit the data with spatial error model to account for the spatial interaction effect using generalized method of moments estimation.^47,48,49,50^ The spatial error model helped capture the spatial spillover in the outcome of mortality rates that was associated with potential omitted variables, while recognizing that county level may not necessarily capture the exact right scale for the regional pattern.^41^

We reported white SEs for all OLS estimates and heteroskedasticity-robust SEs for all spatial error models. All models were implemented in GeoDaSpace, version 1.2.

## Results

Among the 3142 counties included in this study, 531 were identified as concentrated longitudinal-impact counties. Of these counties, 347 (11.0%) were identified as concentrated longitudinal-impact counties with the largest share of Black or African American population among US counties (n = 74 997 322), 198 (6.3%) were concentrated longitudinal-impact counties with the largest share of Hispanic or Latinx population among US counties (n = 98 493 610), and 33 (1.1%) were concentrated longitudinal-impact counties with the largest share of non-Hispanic White population among US counties (n = 1 085 094). Another 2611 counties (83.1%; n = 190 652 685) were not identified as concentrated longitudinal-impact counties. A total of 489 254 COVID-19-related deaths were reported. We stratified mortality rates, SDOH indexes, and structural factors by concentrated longitudinal-impact counties for each racial or ethnic group (Table 1). Density plots (eFigure 3 in the Supplement) illustrate the variation in community characteristics, including mortality rates, SDOH indexes, and structural factors.

**Table 1.** COVID-19 Mortality Rates, Social Determinants of Health Indexes, and Structural Factors by Concentrated Longitudinal-Impact Counties

Variable	Median (IQR)					*P* value^b^
	All	Black or African American	Hispanic or Latinx	Non-Hispanic White	Other^a^	
Counties, No. (%)^c^	3142 (100)	347 (11.0)	198 (6.3)	33 (1.1)	2611 (83.1)	NA
Total population, No.	324 697 795	74 997 322	98 493 610	1 085 094	190 652 685	NA
Total deaths, No.	489 254.6	138 314.9	195 852.5	2586.1	231 009.1	NA
County population	25 740.00 (10 952.00 to 67 866.00)	39 532.00 (20 240.50 to 131 513.50)	152 947.50 (37 141.00 to 535 209.25)	24 926.00 (17 695.00 to 40 884.00)	22 782.00 (9426.50 to 58 344.50)	<.001
No. of deaths	39.00 (14.10 to 95.90)	94.40 (52.40 to 258.60)	308.30 (88.97 to 931.35)	67.00 (47.30 to 85.90)	29.90 (11.20 to 73.20)	<.001
No. of deaths per 100 000 population	146.66 (86.77 to 215.46)	231.43 (181.80 to 289.14)	218.84 (173.31 to 287.39)	251.79 (223.58 to 290.50)	128.55 (75.66 to 191.72)	<.001
No. of days in the top quintile of mortality rate	61.00 (32.00 to 98.00)	128.00 (113.50 to 154.50)	124.00 (111.25 to 147.75)	114.00 (107.00 to 124.00)	51.00 (28.00 to 77.00)	<.001
% Aged 18-64 y	58.89 (56.72 to 61.08)	60.04 (58.47 to 62.10)	59.35 (57.38 to 62.14)	58.14 (56.97 to 59.24)	58.72 (56.34 to 60.95)	<.001
% Aged ≥65 y	18.45 (15.80 to 21.24)	16.73 (14.93 to 18.66)	15.55 (13.61 to 17.53)	19.77 (19.08 to 20.58)	18.90 (16.24 to 21.72)	<.001
% Urban	0 (0 to 18.25)	0 (0 to 81.50)	75.00 (0 to 95.00)	0	0	<.001
% Suburban	15.00 (0 to 50.00)	20.00 (0 to 54.00)	11.00 (2.00 to 50.00)	17.00 (0 to 50.00)	14.00 (0 to 50.00)	.03
% Rural	50.00 (8.00 to 100.00)	32.00 (1.00 to 73.00)	6.00 (1.00 to 37.50)	75.00 (40.00 to 100.00)	62.00 (15.00 to 100.00)	<.001
Socioeconomic advantage index	0.59 (–0.21 to 1.28)	–0.64 (–1.16 to –0.16)	–0.47 (–1.56 to 0.18)	0.98 (0.45 to 1.25)	0.80 (0.12 to 1.40)	<.001
Limited mobility index	–0.50 (–1.05 to 0.05)	–0.76 (–1.18 to –0.29)	0.05 (–0.46 to 0.74)	–0.75 (–1.18 to –0.33)	–0.49 (–1.05 to 0.05)	<.001
Urban core opportunity index	–0.68 (–0.96 to –0.37)	–0.81 (–1.03 to –0.48)	–0.48 (–0.80 to 0)	–0.95 (–1.03 to –0.69)	–0.66 (–0.95 to –0.37)	<.001
Mixed immigrant cohesion and accessibility index	–0.28 (–0.63 to 0.09)	0.02 (–0.32 to 0.37)	–0.43 (–0.95 to 0.08)	–0.34 (–0.52 to –0.11)	–0.30 (–0.65 to 0.05)	<.001
Income inequality	4.40 (4.00 to 4.90)	5.10 (4.70 to 5.60)	4.60 (4.30 to 5.10)	4.10 (3.80 to 4.40)	4.30 (4.00 to 4.70)	<.001
% Without internet access	19.46 (14.77 to 25.16)	25.20 (18.30 to 32.08)	17.35 (12.61 to 23.03)	21.36 (17.92 to 25.51)	19.08 (14.57 to 24.34)	<.001
% Without insurance	11.00 (7.00 to 14.00)	13.00 (10.00 to 16.00)	14.50 (9.00 to 20.00)	8.00 (7.00 to 9.00)	10.00 (7.00 to 14.00)	<.001
Ratio of population to primary care physicians	2003.00 (1372.00 to 3001.00)	2110.00 (1440.00 to 2997.50)	1788.00 (1396.00 to 2387.00)	2372.00 (1988.00 to 2841.00)	1997.00 (1346.00 to 3025.00)	.04
Rate of preventable hospital stays	4710.00 (3613.00 to 5802.50)	5721.00 (5005.00 to 6637.50)	4786.50 (3920.50 to 5511.50)	4808.00 (4315.00 to 6709.00)	4530.00 (3472.75 to 5664.00)	<.001
% Severe housing problems	13.00 (11.00 to 16.00)	16.00 (14.00 to 19.00)	17.00 (14.00 to 20.00)	11.00 (10.00 to 12.00)	13.00 (11.00 to 15.00)	<.001

Abbreviation: NA, not applicable.

^a^ Other included counties that were not identified as concentrated longitudinal-impact counties.

^b^ *P* value was calculated with 1-way analysis of variance.

^c^ A small number of counties (n = 47) were identified as concentrated longitudinal-impact counties with both large Black or African American population and large Hispanic or Latinx population compared to other counties.

By design, all concentrated longitudinal-impact counties had significantly higher COVID-19 mortality rates than other counties during the study period. The median (IQR) deaths per 100 000 population were 231.43 (181.80-289.14) in a concentrated longitudinal-impact county with a large Black or African American population compared with other counties, 218.84 (173.31-287.39) in a concentrated longitudinal-impact county with a large Hispanic or Latinx population compared with other counties, and 251.79 (223.58-290.50) in a concentrated longitudinal-impact county with a large non-Hispanic White population compared with other counties. In contrast, the median (IQR) mortality rate in non–concentrated longitudinal-impact counties was 128.55 (75.66-191.72) per 100 000 population. Although all of the concentrated longitudinal-impact counties were disproportionately affected by COVID-19, the outcomes stratified by their populations were not the same.

First, although the 33 concentrated longitudinal-impact counties with a large non-Hispanic White population compared with other counties had a higher mortality rate per 100 000 population, many more counties were identified as having Black or African American and Hispanic or Latinx concentrations, indicating that concentrated longitudinal-impact counties with large Black or African American and Hispanic or Latinx populations compared with other counties had higher aggregated COVID-19 mortality than all concentrated longitudinal-impact counties with a large non-Hispanic White population compared with other counties (total number of deaths: 138 314.9 and 195 852.5 vs 2586.1). Second, many concentrated longitudinal-impact counties with the largest shares of Black or African American (233 of 347 [67.1%]) and Hispanic or Latinx (110 of 198 [55.6%]) populations among US counties had a higher percentage of working-age people than the national median level. In contrast, 26 of 33 concentrated longitudinal-impact counties with a large non-Hispanic White population compared with other counties (78.8%) had a higher percentage of people aged 65 years or older compared with the national median. Third, 23 of 33 concentrated longitudinal-impact counties with a large non-Hispanic White population compared with other counties (69.7%) were located in rural areas, whereas a large proportion of the concentrated longitudinal-impact counties with a large Hispanic or Latinx population compared with other counties (114 of 198 [57.6%]) were mainly located in urban areas. Concentrated longitudinal-impact counties with a large Black or African American population compared with other counties were a mixture of rural, suburban, and urban areas.

These concentrated longitudinal-impact counties also had substantial variation in SDOH indexes. A large proportion of concentrated longitudinal-impact counties with a large Black or African American population compared with other counties had socioeconomic advantage index (318 of 347 [91.6%]), limited mobility index (221 of 347 [63.7%]), and urban core opportunity index (211 of 347 [60.8%]) values that were lower than the national median values. Meanwhile, in most concentrated longitudinal-impact counties with a large Hispanic or Latinx population compared with other counties, the socioeconomic advantage index (170 of 198 [85.9%]) and mixed immigrant cohesion and accessibility index (115 of 198 [58.1%]) were lower than the national median, but the urban core opportunity index was higher (131 of 198 [66.2%]). A high urban core opportunity index may reflect a higher risk of COVID-19 infection through greater potential workplace exposure given that the index features urbanized populations with more working opportunities. In contrast, most concentrated longitudinal-impact counties with a large non-Hispanic White population compared with other counties had limited mobility index (23 of 33 [69.7%]) and urban core opportunity index (25 of 33 [75.8%]) values that were lower than the national median values. This observation was consistent with the finding that concentrated longitudinal-impact counties with a large non-Hispanic White population compared with other counties were mostly located in rural areas and had higher percentages of residents aged 65 years or older.

In addition to these SDOH indexes, other social factors were uniquely associated with COVID-19 mortality across the different concentrated longitudinal-impact counties. Most concentrated longitudinal-impact counties with a large Black or African American population compared with other counties (297 of 347 [85.6%]) had higher income inequality than the national median. Many concentrated longitudinal-impact counties with large Black or African American (227 of 347 [65.4%]) and Hispanic or Latinx (130 of 347 [65.7%]) populations compared with other counties also had a higher-than-the-national-median percentage of younger people (<65 years) without insurance. Twenty-four of 33 concentrated longitudinal-impact counties with a large non-Hispanic White population compared with other counties (72.7%) had a higher ratio of residents to primary care physicians than the national median ratio. Most concentrated longitudinal-impact counties with a large Black or African American population compared with other counties (281 of 347 [81.0%]) showed greater preventable hospital stays than the national median. Severe housing problem rates were higher than the national median level in concentrated longitudinal-impact counties with large Black or African American (266 of 347 [76.7%]) and Hispanic or Latinx (170 of 198 [85.9%]) populations compared with other counties. In addition, most concentrated longitudinal-impact counties with large Black or African American (244 of 347 [70.3%]) and non-Hispanic White (21 of 33 [63.6%]) populations compared with other counties had higher percentages of households without access to the internet.

**Figure 2.**
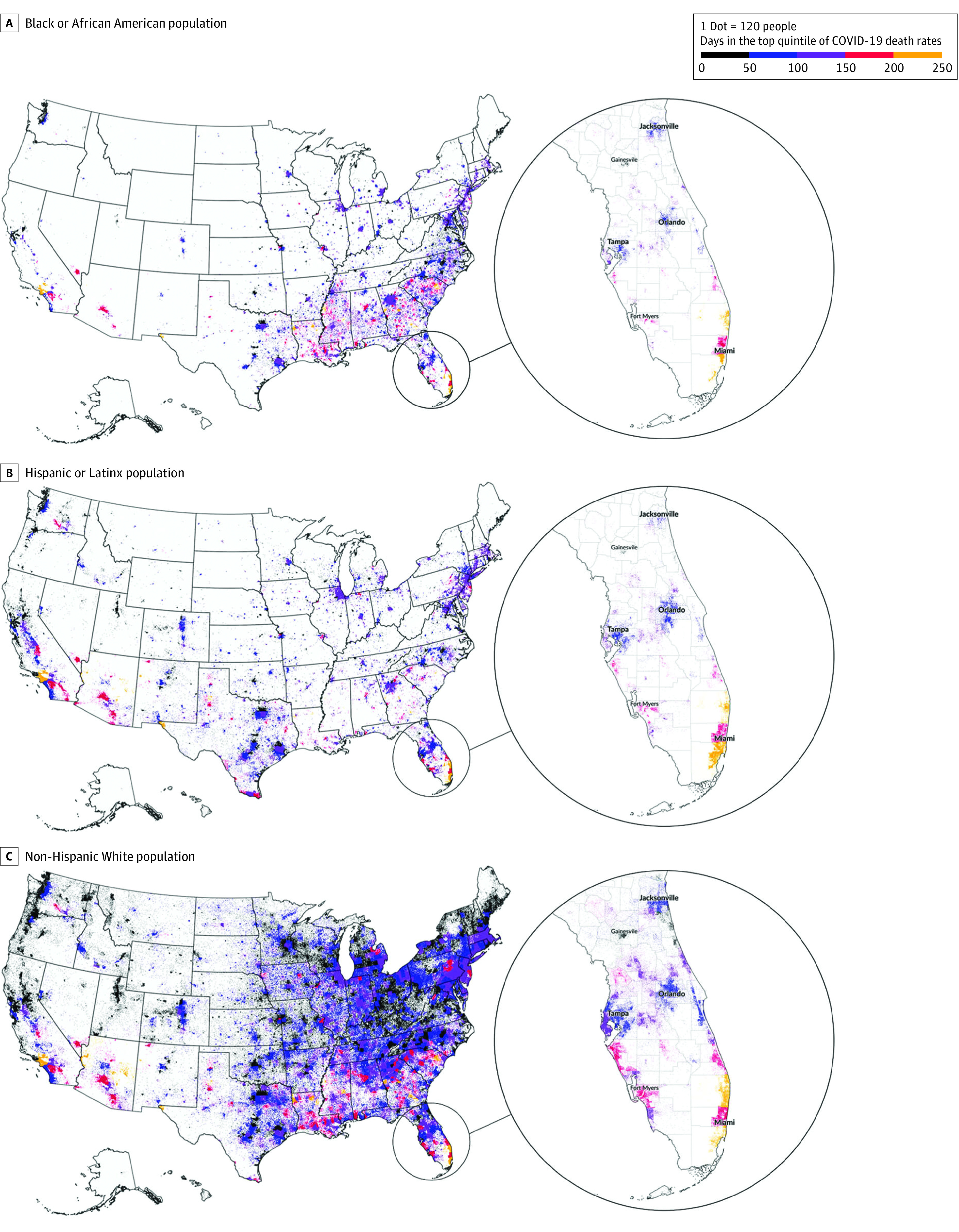
Dot Density Visualization of County-Level COVID-19 Deaths by American Community Survey (ACS) Race and Ethnicity Dots were based on 2019 ACS 5-year population estimates. Maps were generated in QGIS, version 3.12.3.

**Figure 3.**
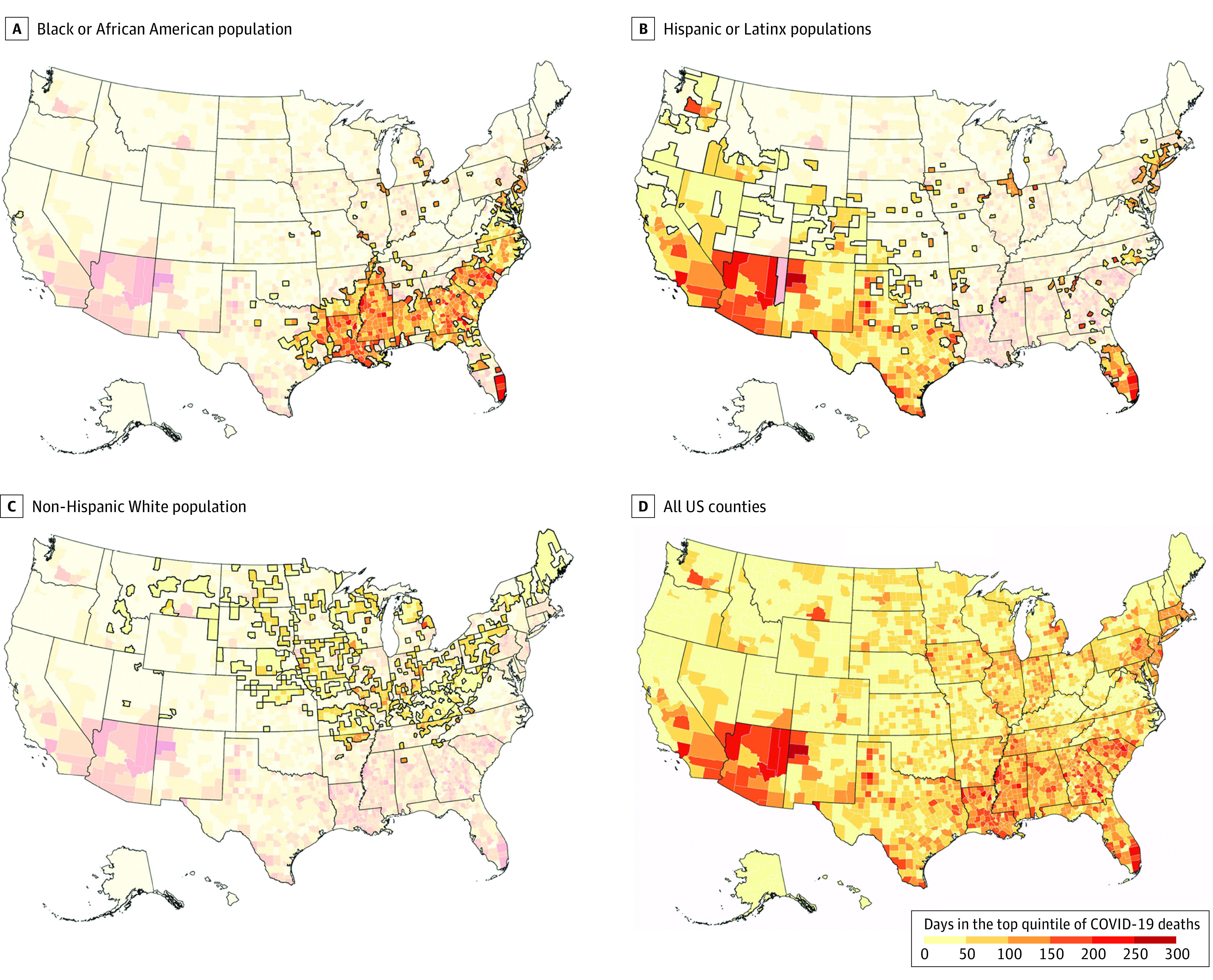
Days in the Top Quintile of COVID-19 Deaths by Racial and Ethnic Group in Top Quintile of Population

On dot density maps (Figure 2), we observed the concentrations of Black or African American populations predominantly across the South and metropolitan counties, many of which had high COVID-19 rates, and Hispanic or Latinx communities that were located in geographically dispersed counties had similarly high rates. In addition, given that the dots indicated a total number of people, it follows that non-Hispanic White populations, which are substantially greater in number in the overall US population across much of the country, were present in many counties that appeared to have a lower number of days in the top COVID-19 death quantile. The choropleth maps (Figure 3) further confirmed these patterns by explicitly identifying concentrated longitudinal-impact counties. Concentrated longitudinal-impact counties with a large Black or African American population compared with other counties were found across the Southeast, representing a range of urban and rural counties, as well as metropolitan areas across the country (eg, Cook County, Illinois, and New York City counties). Concentrated longitudinal-impact counties with a large Hispanic or Latinx population compared with other counties were primarily clustered in the Southwest, representing a mix of urban and suburban counties. Concentrated longitudinal-impact counties with a non-Hispanic White population were found across the Midwest and Appalachia geographic areas of the US.

Both OLS estimates (eTable 2 in the Supplement) and the spatial error models (Table 2) provided statistical evidence for spatial heterogeneity among rural, suburban, and urban areas (global Chow test result in OLS, 140.19; *P* < .001; global Chow test in spatial error model: 205.22; *P* < .001), with most factors having significantly different coefficients across different spatial regimes. According to the Chow test results, this finding was especially true for factors associated with preventable hospital stays (11.96; *P* = .003), socioeconomic advantage index (23.10; *P* < .001), and limited mobility index (19.73; *P* = .001). The models also explained the most variance in COVID-19 mortality rates in urban areas (adjusted *R*^2^ = 0.35) and the least variance in rural areas (adjusted *R^2^* = 0.13). Evidence for spatial dependence was found (coefficient [SE], 0.70 [0.02]; *P* < .001).

**Table 2.** Spatial Error Model With Heteroskedasticity-Robust SEs Associated With COVID-19 Mortality Rate^a^

Factor	Rural (n = 1619)		Suburban (n = 689)		Urban (n = 659)		Chow test	
	Coefficient (SE)	*P* value	Coefficient (SE)	*P* value	Coefficient (SE)	*P* value	Value	*P* value
Constant	67.97 (12.51)	<.001	102.89 (13.50)	<.001	84.34 (14.21)	<.001	4.67	.10
Group quarter rate	0.001 (0.0006)	.06	–0.00002 (0.001)	.79	–0.002 (0.001)	.11	5.66	.06
% Without internet access	2.43 (0.46)	<.001	2.97 (0.56)	<.001	5.83 (0.81)	<.001	14.45	< .001
Preventable hospital stays	0.008 (0.002)	<.001	–0.0002 (0.002)	.93	0.003 (0.002)	.12	11.96	.003
Socioeconomic advantage index	–1.88 (3.20)	.56	–21.60 (3.55)	<.001	–4.24 (2.79)	.13	23.10	<.001
Limited mobility index	11.05 (3.80)	.004	–10.65 (4.17)	.01	–3.18 (4.10)	.44	19.73	<.001
Urban core opportunity index	–11.37 (6.78)	.09	–1.37 (8.28)	.87	5.92 (4.35)	.17	5.14	.08
Mixed immigrant cohesion and accessibility index	–4.29 (5.10)	.40	–0.63 (6.35)	.92	–23.38 (6.06)	<.001	9.93	.007
Delay of mask mandate	–0.01 (0.02)	.72	–0.002 (0.02)	.95	0.03 (0.02)	.20	2.74	.25

^a^ The model used generalized method of moments to estimate the coefficients. 2967 counties in the continental US are included in this model because SDOH indexes are only available for these counties.

First, places with more limited internet access had higher mortality in all spatial regimes (coefficient [SE], rural: 2.43 [0.46], *P* < .001; suburban: 2.97 [0.56], *P* < .001; urban: 5.83 [0.81], *P* < .001). Second, in rural areas, the measure of preventable hospital stays was associated with mortality rates (coefficient [SE], 0.008 [0.002]; *P* < .001). The mask mandate policy variable, which was statistically significant in OLS estimates for urban areas (coefficient [SE], 0.04 [0.02]; *P* = .01), was not significant in the spatial error model (coefficient [SE], 0.03 [0.02]; *P* = .20).

The SDOH indexes also showed distinct patterns in different spatial regimes. For the socioeconomic advantage index, greater vulnerability was associated with higher COVID-19 mortality rates in suburban areas (coefficient [SE], –21.60 [3.55]; *P* < .001). The limited mobility index was associated with COVID-19 mortality in rural areas (coefficient [SE], 11.05 [3.80]; *P* = .004), but it was inversely associated with COVID-19 mortality in suburban areas (coefficient [SE], –10.65 [4.17]; *P* = .01). These observations suggest that higher COVID-19 mortality rates were associated with rural areas with younger populations and/or fewer persons with disabilities, in contrast to suburban areas with older populations and/or more people with disabilities. The mixed immigrant cohesion and accessibility index was inversely associated with COVID-19 mortality in urban areas (coefficient [SE], –23.38 [6.06]; *P* < .001), which suggests that the higher the number of immigrant populations with traditional family structures, multiple accessibility stressors, and housing overcrowding, the higher the COVID-19 mortality rates.

## Discussion

We believe this study contributes new insights about the associations between SDOH and the racial and spatial disparities in COVID-19 mortality rates in the US. We found that, for Black or African American, Hispanic or Latinx, and non-Hispanic White populations, different dimensions of SDOH were uniquely associated with each group’s disproportionate burden of COVID-19 mortality. Furthermore, we found statistical evidence that various SDOH dimensions operated distinctly across rural, suburban, and urban areas to shape COVID-19 mortality. The ESDA approach allowed us to detect this heterogeneous process while accounting for the interdependence between counties. To our knowledge, this analysis was the first to apply the ESDA approach to COVID-19 mortality across racial and ethnic groups and in urban and rural contexts.

We believe this study also extends the findings from previous studies on structural health inequity by focusing on SDOH and structural factors that characterize systemic disadvantages experienced by racial and ethnic minority groups. To this end, we intentionally avoided treating race and ethnicity as preexisting conditions or as explanatory variables on the right-hand side in regression models.^51^ Instead, we used a 2-step approach: first, we explored the SDOH of places in which each racial and ethnic group experienced high COVID-19 mortality (which we termed as concentrated longitudinal-impact counties), and then we examined the associations between SDOH and COVID-19 mortality in different spatial regimes (rural, suburban, and urban). In the initial step of analyzing concentrated longitudinal-impact counties, we found that those counties with a large non-Hispanic White population were composed of more rural counties with significantly smaller total populations compared with the more urban, more densely populated counties with a high concentration of Black or African American and Hispanic or Latinx groups. This contrast was further validated in the second step of the spatial regression analysis, which revealed that different dimensions of SDOH were associated with high COVID-19 mortality rates across rural, suburban, and urban areas. These results underscore how place and people intersect within multifaceted power structures that produce and reproduce inequity in health outcomes.^52,53,54,55^

In urban areas, internet access and the mixed immigrant cohesion and accessibility index were important factors in mortality rates. Consistent with previous research that found that severe housing problems were associated with higher COVID-19 incidence and mortality,^56^ this study further highlighted this association in urban areas and connected it with our finding that most concentrated longitudinal-impact counties with a large Hispanic or Latinx population compared with other counties were located in urban areas. Moreover, we found that these counties had a high percentage of working-age people without health insurance and who were more likely to be exposed to COVID-19 infection. This finding is consistent with previous findings in Latinx adults who were more often at risk for contracting COVID-19 because of work requirements and hesitant about going to a hospital because of economic and immigration concerns.^57^

In suburban areas, higher mortality was associated with lower socioeconomic advantage index and limited mobility index, indicating higher poverty rates in these areas along with higher percentages of older adults and/or people with a disability. More research is needed to understand the mechanisms regarding how SDOH contributes to COVID-19 mortality and/or other health outcomes in suburban areas.

In rural areas, higher COVID-19 mortality was associated with more preventable hospital stays and higher limited mobility index. The preventable hospital stay measure considered both quality and access^58^; the significant association thus highlighted the critical role of access in high-quality health care in rural areas. The association between limited mobility index and COVID-19 mortality was unexpected. A possible explanation for this association is that the limited mobility index captures a potential higher mobility among those with less physical restrictions from age or disability because a low limited mobility index characterizes a high proportion of adults 65 years or older and people with disabilities. We found that most concentrated longitudinal-impact counties with a large non-Hispanic White population compared with other counties had a large number of older adults living in rural areas with limited access to health care. Given the opposite coefficients of the limited mobility index in rural vs suburban regime, future research should examine (1) the mechanism through which older populations are vulnerable to COVID-19 and (2) the association between mobility (measured as movement or traveling) and the spread of COVID-19. A recently published study has started to discuss higher COVID-19 mortality rates in rural areas,^59^ but the underlying mechanisms are still understudied. The results of the present study shed a light on this important area for future research.

Furthermore, internet access was a significant factor in all communities, an observation that extends similar findings from previous work.^26^ Adopting an asset-based approach,^60,61^ we believe this finding suggests that more awareness is needed about the essential asset of technological access to reliable information, remote work, schooling opportunities, resource purchasing, and/or social community. Populations with limited internet access remain understudied and are often excluded in pandemic research.

We also showed statistical evidence for spatial dependence. The mask mandate policy factor was no longer significant after we accounted for spatial heterogeneity and spatial dependence. Although other studies have reported conflicting findings regarding the association between mask mandate and COVID-19 mortality rates,^62,63^ the results of this study suggest a possibility that the mask mandate has implications at a regional level rather than a county level.

Clearly, SDOH dimensions matter for health outcomes, but the results of this study add nuance to this assumption by demonstrating that SDOH potentially shape health in unique ways, depending on a community’s rural and urban contexts as well as its racial and ethnic makeup. We found that non-Hispanic White populations in rural areas and Hispanic or Latinx populations in urban areas were especially vulnerable to COVID-19 mortality, whereas Black or African American populations across rural and urban contexts fared poorly (in terms of mortality rate) during the first year of the pandemic. For urban, rural, and suburban communities, some dimensions of SDOH seemed to be more consequential for COVID-19 mortality rates, pointing to the social levers that might play the biggest role in moving the needle on population health in different types of communities. For future public health interventions and policy proposals, this analysis offers one way to apply a chisel rather than a hammer to identifying, prioritizing, and tackling social factors associated with deeply entrenched health inequities across racial and ethnic groups and spaces.

Understanding health inequities is also important for developing and implementing equitable, place-based interventions. This study calls for similar, future research to guide policies and programs at the county or regional level. A more complex model that uses geospatial and temporal patterns to reflect spatial effects and dynamic community aspects (eg, changes in policies, unemployment), in addition to existing SDOH measures and human behaviors, is needed for future analysis.

### Limitations 

This study has several limitations. First, we excluded analyses for other racial and ethnic groups, such as American Indian and Alaska Native and Asian people, because of the small proportion of these populations in the data set. However, research that includes or focuses on these groups is warranted given the disproportionate COVID-19 burden that these groups have also experienced.^64,65^ Second, this cross-sectional study used ESDA, and thus the results should not be interpreted as causal. Additional longitudinal analyses are needed to evaluate the implications of SDOH for racial and spatial disparities in COVID-19 mortality. Third, the county-level scale may not capture the full picture of the affected populations, and further research is needed when race-disaggregated data become available at granular spatial scales.

Fourth, although we attempted to capture a more persistent and stable pattern by including the data from the entire first year of the COVID-19 pandemic, we acknowledge the potential confounding factor of vaccines being available at the end of 2020. To check this, we ran the analysis without the first 2 months of 2021 and included the results in eTable 3 in the Supplement. The results did not change substantially, and most findings still hold. In addition, this analysis of concentrated longitudinal-impact counties leveraged information about counties that are outliers in terms of racial and ethnic group concentration and mortality. Further investigations should be conducted into whether similar patterns hold in counties with mixed demographic characteristics and high rates of death from COVID-19. In addition to the spatial perspective taken in this study, future research could adopt more sophisticated approaches that relax the identical, independently distributed assumptions to provide better comparisons to epidemiologic classics. Fifth, we primarily analyzed SDOH barriers that were associated with vulnerability to COVID-19 mortality, but future analyses would benefit from an asset-based approach that identifies protective factors and policies that mitigate a community’s COVID-19 risk, such as social safety net spending and community cohesion. Such work could be valuable in identifying communities that have been able to minimize COVID-19 deaths and could inform future policies to tackle health inequities.

## Conclusions

This cross-sectional study found that SDOH measures were associated with spatial and racial inequity in COVID-19 mortality rates in the US. This association varied for Black or African American, Hispanic or Latinx, and non-Hispanic White populations and in different rural and urban contexts. To address health inequities and to guide policies and programs at the county or regional level, future study is needed into the different dimensions and regional patterns of SDOH.

## Notes

Supplement. **eFigure 1.** State-Level COVID-19 Deaths Versus State-Level Population by Race/Ethnicity, Crude and Age Standardized **eFigure 2.** Explanatory Graphic of Queen Contiguity Weights **eFigure 3.** Density Plots of Health Factor, SDOH Index, Demographic and Place Variables by CLICs for Each Race and Ethnicity **eTable 1.** Social Determinants of Health Indices and Structural Factors **eTable 2.** Model Using Ordinary Least Squares With White Errors Associated With COVID-19 Mortality Rate **eTable 3.** Spatial Error Model with HET Standard Errors Associated With COVID-19 Mortality Rate (Excluding January and February 2021)
eReferences
