# Error in the Abstract and Methods

**DOI:** 10.1001/jamanetworkopen.2022.12488

**Published:** 2022-04-25

In the Original Investigation titled “Assessment of Structural Barriers and Racial Group Disparities of COVID-19 Mortality With Spatial Analysis,”^1^ published March 4, 2022, one of the exposures, access to broadband internet, listed in the Abstract and Methods section was incorrect. The correct exposure is access to internet. This article has been corrected.^1^
