# Supplementary material for: Assessment of Structural Barriers and Racial Group Disparities of COVID-19 Mortality With Spatial Analysis
Source: JAMA Netw Open. 2022 Mar 4;5(3):e220984. doi: 10.1001/jamanetworkopen.2022.0984 (PMC8897755; doi:10.1001/jamanetworkopen.2022.0984)
Supplement: Supplement. — eFigure 1. State-Level COVID-19 Deaths Versus State-Level Population by Race/Ethnicity, Crude and Age Standardized eFigure 2. Explanatory Graphic of Queen Contiguity Weights eFigure 3. Density Plots of Health Factor, SDOH Index, Demographic and Place Variables by CLICs for Each Race and Ethnicity eTable 1. Social Determinants of Health Indices and Structural Factors eTable 2. Model Using Ordinary Least Squares With White Errors Associated With COVID-19 Mortality Rate eTable 3. Spatial Error Model with HET Standard Errors Associated With COVID-19 Mortality Rate (Excluding January and February 2021) eReferences [file jamanetwopen-e220984-s001.pdf]

Supplementary Online Content

Lin Q, Paykin S, Halpern D, Martinez-Cardoso A, Kolak M. Assessment of structural barriers and racial group disparities of COVID-19 mortality with spatial analysis. *JAMA Netw Open*. 2022;5(3):e220984. doi:10.1001/jamanetworkopen.2022.0984

eFigure 1. State-Level COVID-19 Deaths Versus State-Level Population by Race/Ethnicity, Crude and Age Standardized

eFigure 2. Explanatory Graphic of Queen Contiguity Weights

eFigure 3. Density Plots of Health Factor, SDOH Index, Demographic and Place Variables by CLICs for Each Race and Ethnicity

eTable 1. Social Determinants of Health Indices and Structural Factors

eTable 2. Model Using Ordinary Least Squares With White Errors Associated With COVID-19 Mortality Rate

eTable 3. Spatial Error Model with HET Standard Errors Associated With COVID-19 Mortality Rate (Excluding January and February 2021)

eReferences

This supplementary material has been provided by the authors to give readers additional information about their work.

© 2022 Lin Q et al. *JAMA Network Open.*

eFigure 1. State-Level COVID-19 Deaths Versus State-Level Population by Race/Ethnicity, Crude and Age Standardized

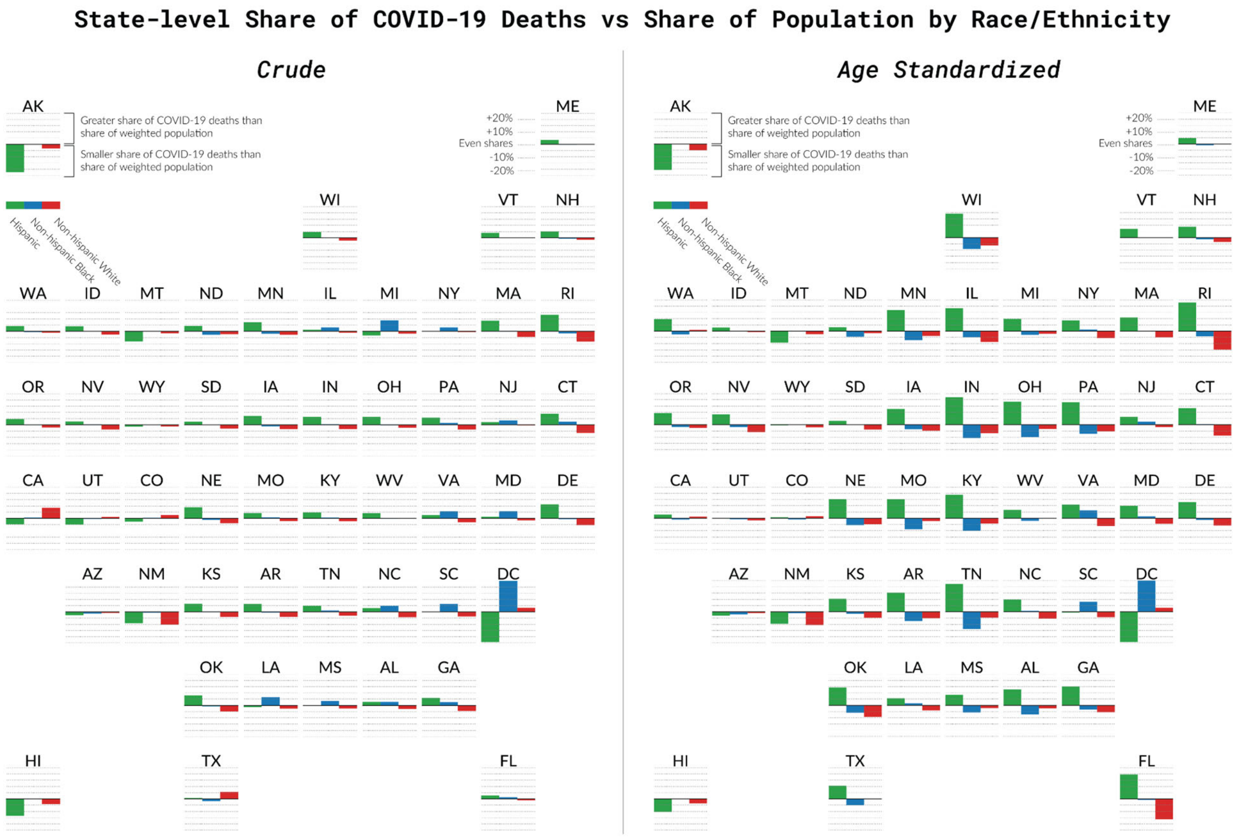

Note. Percentage difference between the share of population of ACS race and ethnicity groups and the share of COVID-19 deaths, aggregated to the nation. If a racial group made up 10% of the population, but experienced 15% of the COVID-19 deaths, the chart would show +.50, or 50% greater share of COVID-19 deaths compared to the share of the population.

© 2022 Lin Q et al. *JAMA Network Open.*

eFigure 2. Explanatory Graphic of Queen Contiguity Weights

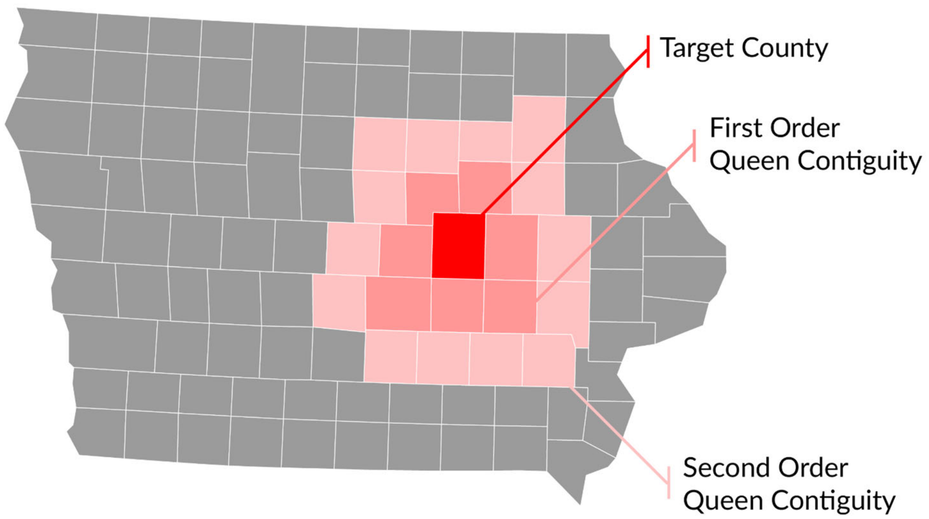

© 2022 Lin Q et al. *JAMA Network Open.*

eFigure 3. Density Plots of Health Factor, SDOH Index, Demographic and Place Variables by CLICs for Each Race and Ethnicity

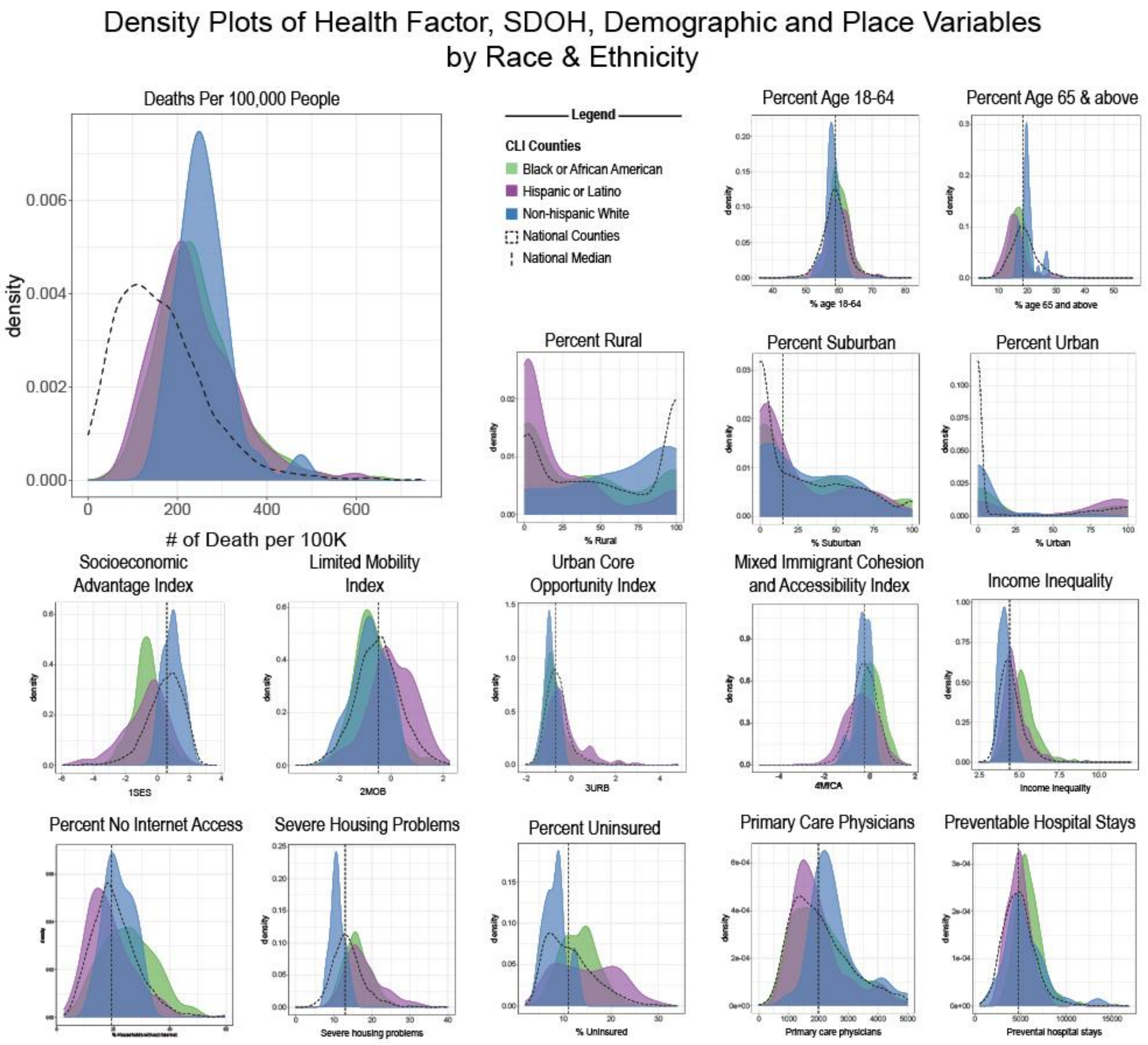

Note: Distributions in the plots are color coded by race: green represents Black population (n = 347, 11.04% of counties); purple represents Hispanic population (n = 198, 6.30% of counties); and blue represents white population (n = 33, 1.05% of counties). The dashed line represents the distribution of all counties, and the vertical line represents the median in the national distribution.

© 2022 Lin Q et al. *JAMA Network Open.*

eTable 1. Social Determinants of Health Indices and Structural Factors

**Measure**	**Description and Data Source**
Socioeconomic advantage index (SES)	Include socioeconomic status factors such as poverty, minority status, educational level, and uninsured; this measure is strongly correlated with the Singh et al[1] areal deprivation index. A low value of this SES index is characterized by a high percentage of population living poverty, with minority status, no high school diploma, and uninsured (low socioeconomic status). (Source: Kolak et al., 2020[2]; Opioid Environment Policy Scan[3]).
Limited mobility index (MOB)	Capture the proportion of older adults and persons with disabilities. A low value of this MOB index is characterized by a high percentage of older adults (aged 65 or over) and people with disabilities. (Source: Kolak et al., 2020[2]; Opioid Environment Policy Scan[3]).
Urban core opportunity index (URB)	Reflects highly urbanized populations experiencing more opportunities and high living costs. A high value of this URB index is characterized by high per capita income, high proportion of renters, high rent burden, and households without a vehicle. (Source: Kolak et al., 2020[2]; Opioid Environment Policy Scan[3])
Mixed immigrant cohesion and accessibility index (MICA)	Features immigrant populations with traditional family structures and multiple accessibility stressors. A lower value of this MICA index is characterized by a high percentage of families with limited English proficiency, older adults, crowded housing, a lack of health insurance, low high school graduation rates, and fewer single parent households. (Source: Kolak et al., 2020[2]; Opioid Environment Policy Scan[3])
Population age distribution	We included percent of working age (age 18 to 64) and percent of senior populations (age 65+). (Source: ACS 2019 5-year estimates[4])
Rural-urban contexts	We classified each county as urban, suburban, or rural if 50% or more of its Census tracts were categorized as such based on their USDA Rural Urban Commuting Area (RUCA) code. (Source: RUCA code[5]; Paykin et al., 2021[6])
Community health factors	We included county-level measures of income inequality (measured as the ratio of household income at the 80th percentile to income at the 20th percentile), uninsured rate, primary care physicians (measured as a ratio of population to primary care physicians), preventable hospital stays (measured as rate of hospital stays for ambulatory-care sensitive conditions per 100K Medicare enrollees), and severe housing problems (measured as percentage of households with at least 1 of 4 housing problems: overcrowding, high housing costs, lack of kitchen facilities, or lack of plumbing facilities). (Source: 2018 County Health Rankings.[7])
Internet access	We measured this as the percentage of households without access to the Internet. (Source: ACS 2019 5-year estimates.[4])
Group quarter population rates	We measured this as the group quarters population per 100K. (Source: ACS 2019 5-year estimates.[4])
Delay in mask mandate policies	The mask mandate variable was calculated by counting how many days a state-level mask mandate was initiated after the county experienced 200 or more

© 2022 Lin Q et al. *JAMA Network Open.*

	new cases per 100K based on the 14-day average. We applied this threshold of 200 as CDC defines this as an indicator for high level community transmission: https://covid.cdc.gov/covid-data-tracker/#county-view. (Source: CDC's Public Mask Mandates dataset.[8])

© 2022 Lin Q et al. *JAMA Network Open.*

eTable 2. Model Using Ordinary Least Squares With White Errors Associated With COVID-19 Mortality Rate

Predictors	Rural Coefficient (SE)	Rural *p*	Suburban Coefficient (SE)	Suburban *p*	Urban Coefficient (SE)	Urban *p*	Chow Test Value	Chow Test *p*
CONSTANT	41.42 (11.78)	<.001	69.79 (13.93)	<.001	6.90 (12.94)	.59	11.07	.004
Group Quarter Rate	**0.001 (0.0005)**	**.01**	0.0006 (0.0008)	.40	**–0.002 (0.0008)**	**.01**	12.62	.002
% No Internet Access	**3.51 (0.42)**	**<.001**	**4.32 (0.56)**	**<.001**	**8.28 (0.74)**	**<.001**	31.93	<.001
Preventable Hospital Stays	**0.01 (0.001)**	**<.001**	0.002 (0.002)	.31	**0.007** (0.002)	**<.001**	8.79	.01
1SES	0.67 (2.84)	.81	**–19.72 (3.16)**	**<.001**	1.60 (2.46)	.52	32.67	<.001
2MOB	**22.40 (3.34)**	**<.001**	4.54 (4.21)	.28	**8.09 (3.59)**	**.02**	13.84	.001
3URB	–2.17 (7.06)	.76	6.70 (9.00)	.46	**29.92 (3.61)**	**<.001**	19.40	<.001
4MICA	–6.30 (5.09)	.22	5.20 (6.15)	.40	**–14.30 (4.45)**	**.001**	6.64	.04
Delay of Mask Mandate	**0.04 (0.02)**	**.02**	0.03 (0.02)	.14	**0.04 (0.02)**	**.01**	0.15	.93
Observations	1619		689		659		Global test	
R^2 / R^2 adjusted	0.135 / 0.131		0.268 / 0.260		0.357 / 0.349		140.19 (p<.001)	
AIC	19554.21		8012.66		7126.78			
Multicollinearity condition number	13.77		12.66		15.60			

Note. All the multicollinearity condition numbers in eTable2 are below 20, indicating no concerning multicollinearity among predictors.

© 2022 Lin Q et al. *JAMA Network Open.*

eTable 3. Spatial Error Model with HET Standard Errors Associated With COVID-19 Mortality Rate (Excluding January and February 2021)

Predictors	Rural		Suburban		Urban		Chow Test	
	Coefficient (SE)	*p*	Coefficient (SE)	*p*	Coefficient (SE)	*P*	Value	*p*
CONSTANT	38.23 (10.22)	<.001	44.06 (10.56)	<.001	43.03 (10.38)	<.001	0.235	.89
Group Quarter Rate	0.0006 (0.0005)	.26	0.0005 (0.0006)	.41	–0.002 (0.001)	.05	5.48	.06
% No Internet Access	**1.91 (0.36)**	**<.001**	**2.37 (0.47)**	**<.001**	**4.67 (0.61)**	**<.001**	16.41	<.001
Preventable Hospital Stays	**0.006 (0.001)**	**<.001**	0.001 (0.002)	.40	0.003 (0.002)	.06	6.53	.04
1SES	0.26 (2.73)	.93	**–13.53 (2.85)**	**<.001**	–3.92 (2.24)	.08	14.59	<.001
2MOB	**10.91 (3.18)**	**<.001**	–1.69 (3.34)	.62	2.32 (3.17)	.46	9.93	.007
3URB	–6.92 (5.66)	.22	3.99 (6.04)	.51	**11.70 (3.48)**	**<.001**	8.80	.01
4MICA	3.47 (4.31)	.42	–0.43 (4.79)	.93	**–13.26 (4.86)**	**.006**	8.49	.01
Delay of Mask Mandate	0.006 (0.02)	.75	0.02 (0.02)	0.28	0.03 (0.02)	.07	1.69	.43
Lamda	**0.68 (Std. Error = 0.02, *p* < .001)**							
Observations	1619		689		659		Global test	
Pseudo R^2	0.128						194.31 (*p*<.001)	

© 2022 Lin Q et al. *JAMA Network Open.*

eReferences

1. Gopal K. Singh. Area Deprivation and Widening Inequalities in US Mortality, 1969–1998 | AJPH | Vol. 93 Issue 7. Accessed September 9, 2021. https://ajph-aphapublications-org.proxy.uchicago.edu/doi/full/10.2105/AJPH.93.7.1137

2. Kolak M, Bhatt J, Park YH, Padrón NA, Molefe A. Quantification of Neighborhood-Level Social Determinants of Health in the Continental United States. *JAMA Netw Open*. 2020;3(1):e1919928. doi:10.1001/jamanetworkopen.2019.19928

3. Paykin S, Halpern D, Lin Q, Menghaney M, Li A, Vigil, R, Bolanos-Gamez, M, Jin, A, Muszynski, A, Kolak M. *GeoDaCenter/Opioid-Policy-Scan: Opioid Environment Policy Scan Data Warehouse*. Zenodo; 2022. doi:10.5281/ZENODO.5842465

4. US Census Bureau. American community survey. Published online 2019.

5. USDA. Rural-Urban Commuting Area Codes | Ag Data Commons. Accessed July 5, 2021. https://data.nal.usda.gov/dataset/rural-urban-commuting-area-codes

6. Paykin S, Menghaney, Moksha, Lin, Qinyun, Kolak, Marynia. Rural, Suburban, Urban Classification for Small Area Analysis. Published online September 2021. http://dx.doi.org/10.13140/RG.2.2.25148.16009

7. How Healthy is your County? | County Health Rankings. County Health Rankings & Roadmaps. Accessed September 7, 2021. https://www.countyhealthrankings.org/county-health-rankings-roadmaps

8. U.S. State and Territorial Public Mask Mandates From April 10, 2020 through July 20, 2021 by County by Day | Data | Centers for Disease Control and Prevention. Accessed September 7, 2021. https://data.cdc.gov/Policy-Surveillance/U-S-State-and-Territorial-Public-Mask-Mandates-Fro/62d6-pm5i

© 2022 Lin Q et al. *JAMA Network Open.*
